# Metabolic Engineering Strategies for Co-Utilization of Carbon Sources in Microbes

**DOI:** 10.3390/bioengineering3010010

**Published:** 2016-02-06

**Authors:** Yifei Wu, Xiaolin Shen, Qipeng Yuan, Yajun Yan

**Affiliations:** 1 State Key Laboratory of Chemical Resource Engineering, Beijing University of Chemical Technology, Beijing 100029, China; hyf-141@163.com (Y.W.); xiaolinshen731@gmail.com (X.S.); 2 BioChemical Engineering Program, College of Engineering, University of Georgia, Athens, GA 30602, USA

**Keywords:** metabolic engineering strategies, co-utilization, lignocellulosic biomass

## Abstract

Co-utilization of carbon sources in microbes is an important topic in metabolic engineering research. It is not only a way to reduce microbial production costs but also an attempt for either improving the yields of target products or decreasing the formation of byproducts. However, there are barriers in co-utilization of carbon sources in microbes, such as carbon catabolite repression. To overcome the barriers, different metabolic engineering strategies have been developed, such as inactivation of the phosphotransferase system and rewiring carbon assimilation pathways. This review summarizes the most recent developments of different strategies that support microbes to utilize two or more carbon sources simultaneously. The main content focuses on the co-utilization of glucose and pentoses, major sugars in lignocellulose.

## 1. Introduction

Microbial production using renewable and economical carbon sources has been highly preferred to address the increasing concerns on the global energy shortage and climate change. Lignocellulosic biomass has become an attractive carbon source for microbial production in recent years, because its hydrolysates substantially offer several simple sugars, such as glucose and pentoses, that can be readily utilized by microorganisms [1]. In addition, other carbon sources in hydrolysates, such as acetate, can also be used for supporting microbial growth and production [2,3]. However, achieving high-yield and cost-effective microbial production of target products from lignocellulosic resources still have challenges from the laboratory scale to the industrial scale, one of which is how to achieve efficient utilization of the mixed sugars. Most microbes consume these mixed sugars sequentially or selectively due to the carbon catabolite repression (CCR), which prolongs the microbial production process and reduces the efficiency of carbon conversion. In order to solve this issue, co-utilization of mixed carbon sources in microbes is highly preferred.

For most microbes, CCR leads to preferential utilization of one carbon source when there are two or more carbon sources in the medium. CCR is an essential regulatory system which can guarantee microbes to obtain specific carbon sources efficiently and survive in competing environments [4]. However, the selective utilization of carbon sources caused by CCR is sometimes a barrier for simultaneous utilization of different carbon sources in bioengineering process. To overcome this barrier, metabolic engineering strategies have been developed against different mechanisms of CCR. This review will summarize the recent efforts devoted to the strategies for eliminating CCR caused by different mixed carbon sources with the emphasis on co-utilization of glucose and pentoses.

## 2. Glucose and Pentoses from Lignocellulosic Biomass

Lignocellulosic biomass is cheap and renewable for bio-based chemical production. The worldwide production of lignocellulose is estimated to be 1.8 × 10^11^ tons/year [5]. Lignocellulose is composed of the heterogeneous complex of cellulose (30%–50%), hemicellulose (15%–35%), and lignin (10%–30%) [6]. Cellulose is a polymer of glucose, while hemicellulose is a heteropolymer of glucose and pentoses, such as xylose and arabinose [7]. Lignin is a complex of polymers of cross-linked phenylpropane units. After hydrolysis of lignocellulose, glucose and pentoses are the predominant sugars as the carbon sources. Co-utilization of glucose and pentoses can be realized via different strategies, depending on the features of microbes (Table 1).

**Figure 1:**
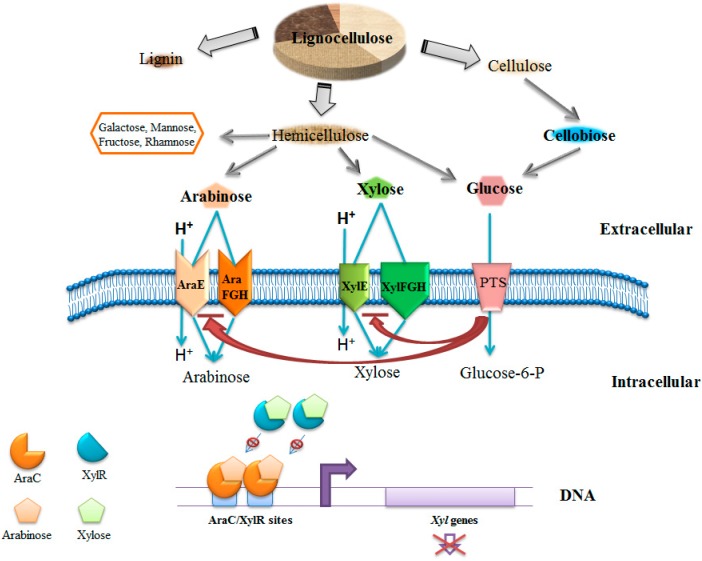
Brief mechanism of CCR among glucose, xylose, and arabinose in *E.coli.* Glucose, xylose, and arabinose are major fermentative carbohydrates from lignocellulose. In the presence of glucose, the CCR induced by PTS inhibits the transportation of xylose and arabinose. In the presence of xylose and arabinose, the transcriptional activators AraC and XylR regulate the uptake of xylose and arabinose. Arabinose-bound AraC displaces xylose-bound XylR from the *xyl* promotor and represses the expression of *xyl* genes. PTS, phosphoenolpyruvate-carbohydrate phosphotransferase system; XylE, xylose-proton symporter; XylFGH, xylose ABC transporters; AraE, arabinose-proton symporter; AraFGH, arabinose ABC transporters.

**Figure 2:**
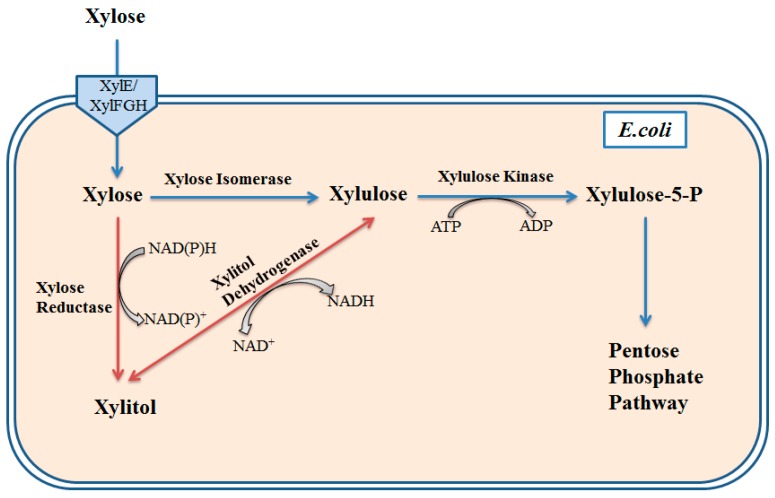
Metabolic engineering of xylitol production in *E. coli*. Xylose is transferred into *E.coli* cells by xylose transporters. Xylose can be converted into xylulose by native xylose isomerase, which is subsequently phosphorylated to xylulose-5-phosphate by xylulose kinase. Xylulose-5-phosphate is an intermediate of the pentose phosphate pathway. Introduction of heterologous xylose reductase or reversible xylitol dehydrogenase can achieve the xylitol production. Xylose reductase reduces xylose to xylitol using NADH or NADPH, and xylitol dehydrogenase reduces xylulose to xylitol using NADH. Blue lines represent the native pathway in *E.coli* and red lines represent the heterologous pathway for xylitol production.

*Escherichia coli*, a widely used industrial production species, possesses CCR that is mainly mediated by inducer exclusion coupled to the phosphoenolpyruvate-dependent sugar phosphotransferase system (PTS) [7,8,9]. Inducer exclusion is a process that the dephosphorylated EIIA^glc^ could inhibit the activity of non-PTS sugar transport system in present of glucose (Figure 1).To disable this mechanism of CCR, *E. coli* PTS disruption strains were constructed, which could consume glucose and xylose simultaneously to produce various polyhydroxyalkanoates (PHAs) [10]. The *ptsG* mutant showed better growth and better utilization than wild-type and the accumulation of short-chain-length PHAs was improved from 7.1% of cellular dry weight (CDW) to 11.5% of CDW after 48 hours [10]. In addition to the deletion of *ptsG*, disruption of *ptsHIcrr-*encoding sugar non-specific protein constituents of the PTS could also relieve the CCR [11]. However, deficiency of PTS can affect the transportation and phosphorylation of glucose because it is the main method for glucose uptake in *E. coli* [12]. To address this problem, the *glf*-encoding glucose facilitator of *Z. mobilis* was integrated into the genome for enhancement of the glucose-utilizing rate. Meanwhile, increasing the activity of the pentose phosphate pathway strengthened the utilization of xylose. With this strategy, the resulting strain could produce 29 g/L ethanol in around 16 h, which accounted for 97% of the theoretical ethanol yield, by consuming glucose and xylose simultaneously [13]. In addition, global transcriptional regulators can also mediate CCR, like the cAMP receptor protein (CRP). Replacement of *E. coli*-native CRP with a cyclic AMP-independent mutant increased the xylose utilization in the presence of glucose. The molar ratio of xylitol was improved after deletion of the *xylB* gene and overexpression of heterologous xylose reductase or xylitol dehydrogenase (Figure 2). By overexpression of xylose reductase from *Candida boidinii*, the concentration of xylitol was about 38 g/L in a fermenter with a 10 L working volume [14]. Among the sugars in lignocellulosic hydrolysates, *E. coli* prefers to consume arabinose than xylose in the absence of glucose. This is because arabinose transcriptional regulator (AraC) can also inhibit xylose transcriptional regulator (XylR), leading to the repression of xylose metabolism (Figure 1). Hence, deletion of the *araC* gene can achieve the co-utilization of xylose and arabinose [15]. Kim *et al.* constructed an evolved strain, GX50 ,containing *araC* deletion and pentose operon constitutive expression, which could be used as a platform strain for co-utilization of glucose and xylose for xylitol production [15]. Another strategy to relieve arabinose-xylose diauxie is to optimize the XylR expression level in *E. coli*. The engineered strain could produce 36% more ethanol than the wild-type in 72 h [16].

**Table 1:** The strategies for co-utilization of glucose and pentoses from lignocellulosic biomass in different microbes.

Microbe	Strategy	Carbohydrates	Product	References
***Escherichia coli***	Inactivation of *ptsG* gene	Glucose and xylose	Ethanol	[13]
	Deletion of *ptsG* gene	Glucose and xylose	Polyhydroxyalkanoates	[10]
	Replacement of native cyclic AMP receptor protein with a cyclic AMP-independent mutant	Glucose and xylose	Xylitol	[14]
	Engineering of *chb* and *asc* operons and adaptive evolution	Cellobiose and xylose	-	[37]
	Expression of *xylR* at the appropriate level	Xylose and arabinose	Ethanol	[16]
	Deletion of *araC*, constitutive expression of genes required for pentose metabolism and adaptive evolution	Glucose and xylose	Xylitol	[15]
	Inactivation of *ptsHIcrr* gene; overexpression of *galP*	Glucose, xylose, and arabinose	Cinnamic and p-hydroxycinnamicacid	[11]
***Saccharomyces cerevisiae***	Expression of the xylose isomerase; Overexpression of *XKS1*, *RPE1*, *RKI1*, *TAL1*, and *TKL1*; Deletion of *GRE3* and *COX4* genes; Adaptive evolution	Glucose and xylose	Ethanol	[38]
	Construction of a growth-based screening system for mutant hexose transporters	Glucose and xylose	-	[19]
	Deletion of d-ribulose-5-phosphate 3-epimerase	Glucose and xylose	Ethanol	[20]
	Maintaining glucose in the useful concentration range in fed-batch reaction	Glucose and xylose	Ethanol	[39]
	Expression of xylose reductase, xylitol dehydrogenase and xylulokinase; Engineering of hexose transporters	Glucose and xylose	Ethanol	[18]
	Evolutionary engineering strategy based on repeated batch cultivation with repeated cycles of consecutive growth	Glucose, xylose, and arabinose	Ethanol	[24]
	Evolutionary engineering via continuous culture using xylose and arabinose as limiting carbon sources	Xylose and arabinose	Ethanol	[23]
	Expression of a cellodextrin transporter, intracellular β-glucosidase and xylose reductase and optimization of the expression	Cellobiose and xylose	Xylitol Ethanol	[22]
	Integration of the fermentation pathways of cellobiose and xylose and an acetic acid reduction pathway	Cellobiose, xylose, and acetic acid	Ethanol	[40]
***Saccharomyces pastorianus***	Co-expression of all three classes of cellulase genes with the *xyl1/xdh1/XKS* genes	Xylose and cellulose	Alcohol	[41]
***Clostridium acetobutylicum***	CcpA mutagenesis	Glucose and xylose	Acetone, Butanol, Ethanol	[25,26]
	Inactivation of *glcG* and overexpression of the rate-limiting steps in xylose pathway	Glucose, xylose, and arabinose	Acetone, Butanol, Ethanol	[27]
***Clostridium sp. Strain BOH3***	Expression of xylose isomerase and xylulokinase	Glucose and xylose	Butanol Riboflavin	[28]
***Clostridium tyrobutyricum***	Overexpression of *xylT*, *xylA*, and *xylB*	Glucose and xylose	n-Butanol	[42]
***Corynebacterium glutamicum***	Expression of *araBAD* operon and/or the *xylA* gene from *Escherichia coli*	Glucose, xylose, and arabinose	Amino Acid	[43]
***Enterococcus mundtii QU 25***	Maintaining the glucose concentration lower than 25 g/L	Glucose and xylose	L-Lactic acid	[44]
***Cryptococcus curvatus***	Decreasing glucose concentration	Glucose, xylose, and cellobiose	Microbial lipid	[45]

*Saccharomyces cerevisiae* is also a potential host for biotechnological production of fuels and chemicals [17]. It can utilize glucose, but not xylose, as a carbon source. Therefore, most strategies usually began with the overexpression of the xylose utilization pathway. The introduction of xylose reductase, xylitol dehydrogenase, and xylulokinase in *S. cerevisiae* could allow the strain to co-utilize glucose and xylose after adjusting the expression of hexose transporter permeases [18]. This strategy was used to produce ethanol, and the affinity of different transporters affected the consumption of sugars and the production of ethanol. The strain with overexpression of the transporter encoded by *HXT1* could achieve the maximal consumption of sugars and ethanol production rate in the glucose and xylose co-fermentation process [18]. The ethanol production was higher than that with either glucose or xylose. However, it cannot consume xylose significantly in a xylose-enriched medium, whereas the *HXT7* permease would be a better choice to allow the xylose uptake rate to be the same as that of glucose [18]. In addition to adjusting the expression of hexose transporter permeases for decreasing glucose repression, the mutations on hexose transporters could also prevent the competitive inhibition from glucose. The mutation sites were designed on hexose transporters in yeast in order to transport xylose rather than glucose [19]. This research demonstrated that the mutant Gal2-N376F can transport xylose with the highest affinity and cannot transport any hexose at all [19]. Furthermore, the deletion of ᴅ-ribulose-5-phosphate 3-epimerase (*RPE1*) is another way to achieve the co-utilization of glucose and xylose for ethanol production [20]. After the xylose utilization pathway was integrated into the genome, *RPE1* was deleted to reduce the flow of carbon from glucose into the pentose phosphate pathway. The ratio of xylose and glucose consumption was about 1:10 during the fermentation by the strain lacking *RPE1* [20]. To avoid the inhibition of glucose and insufficient supply of NAD(P)H for xylose reductase, cellobiose, a disaccharide of glucose, was chosen to co-utilize with xylose. The cellobiose metabolic pathway and xylose reductase were introduced into *S. cerevisiae* for utilization of cellobiose and xylose simultaneously [21]. The engineered strain could produce 19 g/L xylitol with co-fermentation of cellobiose and xylose within 48 h, while the titer of xylitol was only 13 g/L, with sequential utilization of glucose and xylose [21]. When the initial concentrations of the cellobiose and xylose were higher, the titer of the xylitol was higher. By overexpression of NADPH-regenerating enzymes, the productivity of xylitol increased by 37%–63% [21]. Zha *et al.* used the same strategy to realize the co-fermentation of these two sugars. Through the optimization of exogenous gene expression, two sugars had similar consumption rates and the production of xylitol increased by 85.7% in 120 h [22]. Moreover, evolutionary engineering approaches were employed to make strains suitable for co-utilizing the mixed sugars in the medium. After the continuous culture for evolution, the evolved strain that carried the xylose and arabinose pathways could produce ethanol with the yield of 0.29 g per consumed xylose (g) at 120 h [23]. Wisselink *et al.* improved the strain by a novel evolutionary engineering strategy which consists of “repeated batch cultivation with repeated cycles of consecutive growth in three media with different compositions”. The evolved strain had a higher yield of biomass and exhibited a high yield of ethanol, 0.44 g/g of total sugars [24].

In addition, co-utilization of glucose and major pentoses from lignocellulosic biomass has also been applied in other microbes. For the industrial solvent-producing strain *Clostridium acetobutylicum*, the disruption of the gene *ccpA*, which encodes a catabolite control protein, could lead to the simultaneous utilization of glucose and xylose. With this strategy, the engineered strain could produce acetone, butanol, and ethanol with titers of 4.94, 12.05, and 1.04 g/L, respectively, in around 36 h [25]. Based on the CcpA molecular modulation, Wu constructed CcpA mutants to overcome CCR and overexpressed *sol* genes to accelerate the sugar consumption and product formation. Finally, the engineered strain could produce 5.01 g/L acetone, 8.61 g/L butanol, and 0.94 g/L ethanol within 60 h [26]. Another way to release CCR in *C. acetobutylicum* was inactivation of *glcG* gene-encoding enzyme II in PTS [27]. By disruption of the *glcG* gene and overexpression of xylose pathway, the strain could achieve 16.06 g/L as the total titer of acetone, butanol, and ethanol in the mixtures of glucose, xylose, and arabinose. Furthermore, activating xylose pathway or maintaining a low concentration of glucose can also lead to the co-utilization of glucose and pentoses from the lignocellulosic hydrolysates [28].

## 3. Glucose and Galactose from Marine Plant Biomass

In addition to lignocellulosic biomass, marine plant biomass has become into another renewable and cost-effective feedstock for microbial production [29]. Marine plant biomass is basically marine macroalgae, which is a kind of potential feedstock with many advantages. Compared with lignocellulosic biomass, marine macroalgae have higher photon conversion efficiency and carbon dioxide fixation rate. Moreover, marine macroalgae lack the recalcitrant cell wall structure so that the enzymatic hydrolysis proceeds much easier in marine macroalgae than lignocellulosic biomass [29,30]. Especially, the red algae are one of the different types of marine macroalgae. They have high fermentable carbohydrate content, including glucose and galactose, which are released from cellulose and agarose in red algae, respectively [17,31]. CCR is still the obstacle in the co-utilization of glucose and galactose. To bypass the repression of glucose, cellobiose, and galactose were co-fermented by engineered *S. cerevisiae*, in which genes encoding a cellodextrin transporter and an intracellular β-glucosidase were over-expressed [30]. Finally, the cell growth, titer, and yield of ethanol were increased, compared with sequential utilization of glucose and galactose [30]. Since the consumption rate of cellobiose was slow, glucose was co-utilized with galactose in *S. cerevisiae* to produce enantiopure *(2R*, *3R)*-butanediol with 70% of the theoretical maximum yield. The repression of glucose was alleviated after introducing the internal truncated *MTH1* that encodes a transcription factor related to hexose transport [17]. Further improvement on the glucose consumption rate was realized by adaptive evolution for practical use [17]. In addition to *S. cerevisiae*, the galactose metabolic pathway was rebuilt with synthetic components, such as promoters and terminators in *E. coli*, for the co-fermentation of glucose and galactose [32]. The uptake of galactose could increase by 53.1% in the final strain compared with the wild-type strain.

## 4. Glucose and Non-Carbohydrates

Simultaneous utilization of glucose and non-carbohydrates such as glycerol and acetate is another way to achieve highly-efficient biological production. Glycerol is a renewable and abundant feedstock with great availability, low price, and high degree of reduction [33]. The co-utilization of glucose and glycerol can be realized by deletion of the *ptsHIcrr* operon of PTS in *E.coli* [34]. The strain PB12 which could grow fast on glucose was isolated to produce aromatic compounds from glucose and other carbon sources. In the mixture of glucose and glycerol, the yield of aromatic compounds was higher and the titer of byproduct acetate was much lower [34]. Shiue *et al.* constructed an *E. coli* Δ*pgi* Δ*zwf* mutant strain for ᴅ-glucaric acid production. The deletion of genes *pgi* and *zwf* could not only eliminate catabolite repression but also improve the strain’s ability to produce glucose-derived products. As the results, the titer of ᴅ-glucaric acid was higher in the *E. coli* Δ*pgi* Δ*zwf* mutant strain and the yield of ᴅ-glucaric acid on glucose increased by nine-fold in the medium with glucose and glycerol [35]. For the co-utilization of glucose and acetate, acetate could play different roles. To increase the intracellular PEP for aromatics production, the genes *pykA*, *pykF*, and *ppsA* were deleted in an *E. coli ptsHIcrr−* strain. As a consequence, the mutant strain could not grow on either glucose or acetate but could grow in the presence of both carbon sources. Herein, acetate played a role as a gluconeogenic substrate. At last, the yield of total aromatics could increase by four-fold, compared with the control strain [36]. To increase the carbon yield and balance the co-factor, acetate was used as the substrate to generate acetyl-CoA for isobutyl acetate production. The deletion of native genes for conversion of pyruvate to acetyl-CoA, and the introduction of an acetate-assimilating pathway, led to the theoretical maximum carbon yield of isobutyl acetate increased from 67% to 75% and the practical yield improved from 35% to 59% after 120 h of production [2].

## 5. Conclusions and Future Perspectives

The preferential utilization of glucose is one of the major limitations for the co-utilization of carbon sources in microbes. The current metabolic engineering strategies to address this limitation mainly focus on the following aspects: (1) eliminating the CCR caused by glucose transportation; (2) enhancing the efficiency of other carbohydrate transportation; and (3) introducing exogenous genes or pathways to improve the efficiency of co-utilization of carbon sources. Although the mechanisms of CCR in different microbes are various, the principles used to relieve CCR are similar. Overall, co-utilization of carbon sources is essential for low-cost microbial production. The existing strategies can be further combined and optimized to achieve target production based upon the carbon sources to be used.
